# Supplementary material for: Abundant clock proteins point to missing molecular regulation in the plant circadian clock
Source: Mol Syst Biol. 2025 Feb 20;21(4):361–89. doi: 10.1038/s44320-025-00086-5 (PMC11965494; doi:10.1038/s44320-025-00086-5)
Supplement: Supplementary file 11 — Expanded View Figures [file 44320_2025_86_MOESM11_ESM.pdf]

Expanded View Figures

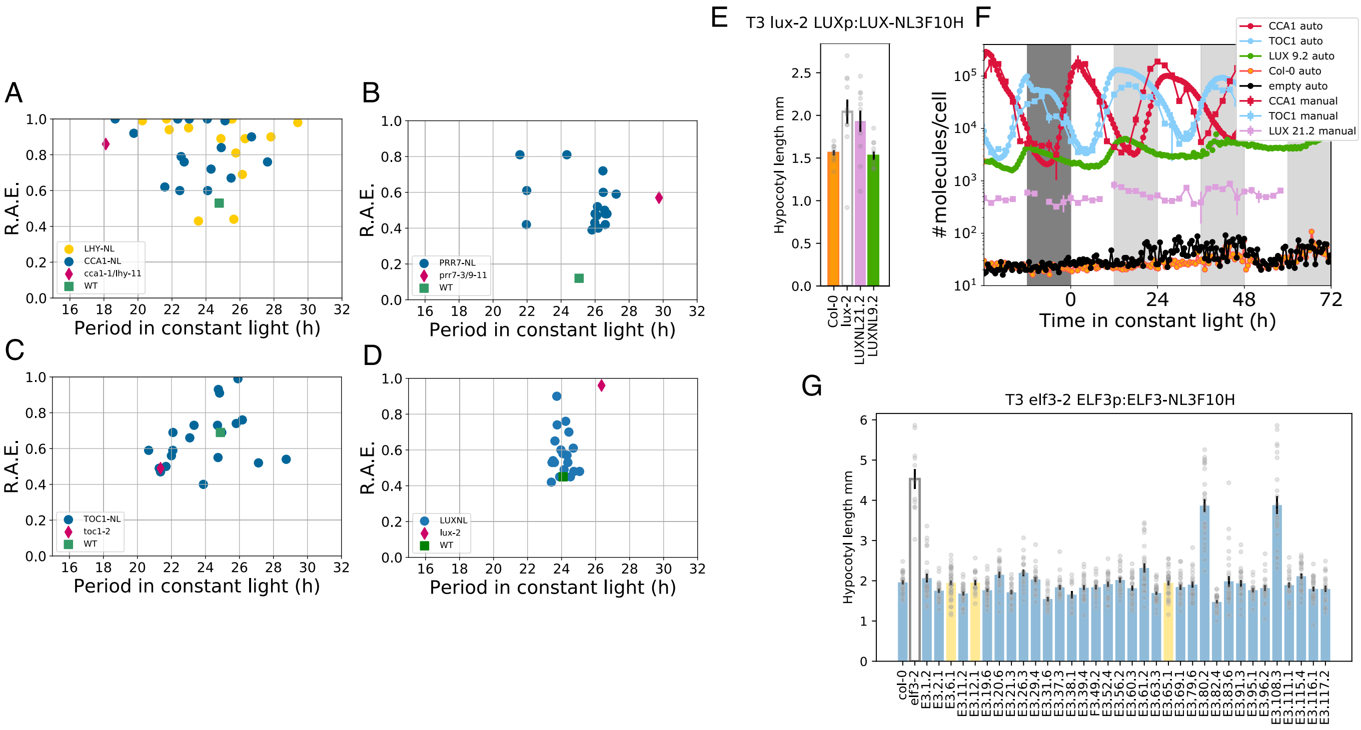

Figure EV1. Reporter fusion constructs rescue clock mutant phenotypes.

Each NanoLUC protein reporter line was selected for the rescue of a clock gene mutant phenotype. (**A–D**) Circadian rhythms in reporter lines were monitored by in vivo imaging of seedlings under constant light, using the firefly LUC transcriptional reporter included in the background of each mutant. Each data point represents the period and relative amplitude error (R.A.E.) of a group of seedlings ($n = 10$) from an independent, single-insert, homozygous line in the T3 generation for the constructs listed, compared to the mutant host (red diamond) and wild type (green square) controls. (**A**) CCA1p:CCA1-NL3F10 (blue) and LHYp:LHY-NL3F10H (yellow) in the mutant background *cca1-1/lhy-11 CCA1p:LUC*. (**B**) PRR7p:PRR7-NL3F10H in *prr7-3/prr9-11 CCR2:LUC*. (**C**) TOC1p:TOC1-NL3F10H in *toc1-2 CCA1p:LUC*. (**D**) LUX2p:LUX-NL3F10H in *lux-2 CAB2:LUC*. (**E–G**) In a second round of selection, (**E**) a further LUX protein reporter line was selected (line 9.2, green), which was complemented to the Col-0 hypocotyl length (orange) whereas the line 21.2 (pink) retained the long hypocotyl phenotype of the *lux-2* mutant parent (open bar). (**F**) In vivo data ('auto') from line 9.2 under LD and LL (green), after the same detrending and rescaling as for CCA1 (red) and TOC1 (blue) (see Methods), suggested an expression level about 7-fold higher than line 21.2 (pink) tested in extracts ('manual'). Col-0 controls (orange) had the same low background signal as an empty well (black). (**G**) the ELF protein reporter lines (yellow) were selected based on the hypocotyl phenotype rescued to match Col-0 (far left, blue) and clearly rescue the long hypocotyl of the *elf3-2* mutant control (open bar).

 © The Author(s)

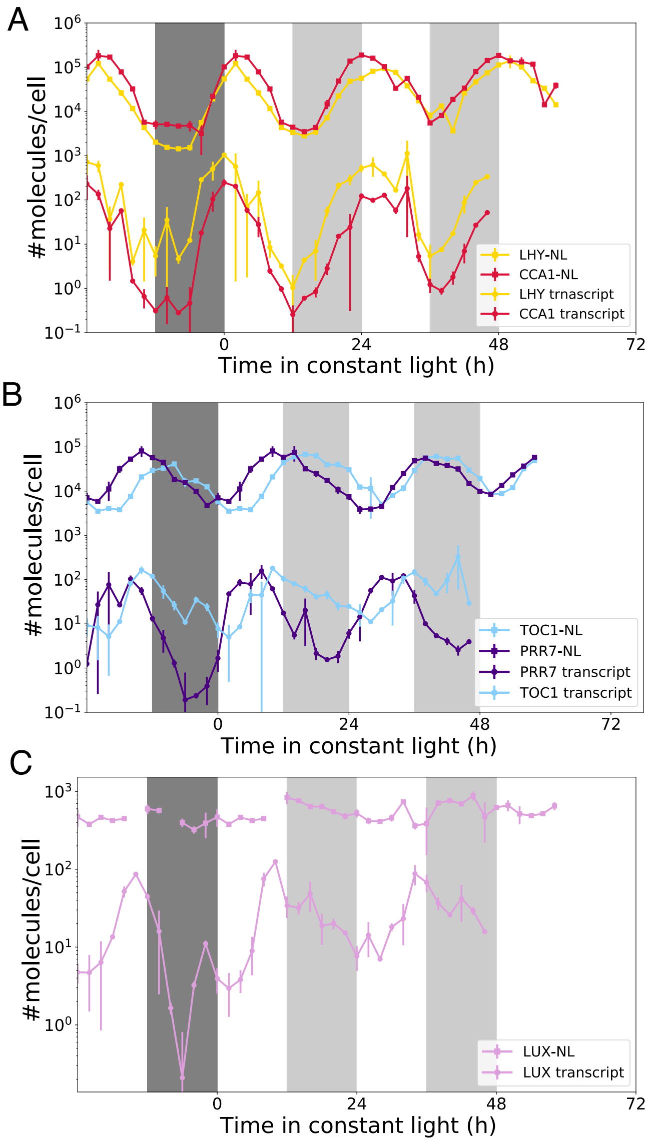

Figure EV2. Timeseries of clock protein copy numbers relative to mRNA.

Reporter protein levels in plant extracts (data as in Fig. 6) were measured in calibrated NanoLUC assays for (**A**) LHY and CCA1, (**B**) PRR7 and TOC1 and (**C**) LUX line 21.2, under a 12L:12D cycle followed by constant light from time 0 h. Note that this LUX line was only partially complemented, see Appendix. Protein levels are compared to RNA levels in the TiMet data set, each in units of molecules per cell. Protein data are means of biological triplicates, RNA data are means of duplicates, error bar = 1 SEM. Light interval, white background; dark interval, dark grey shading; anticipated dark interval during constant light, light grey shading.

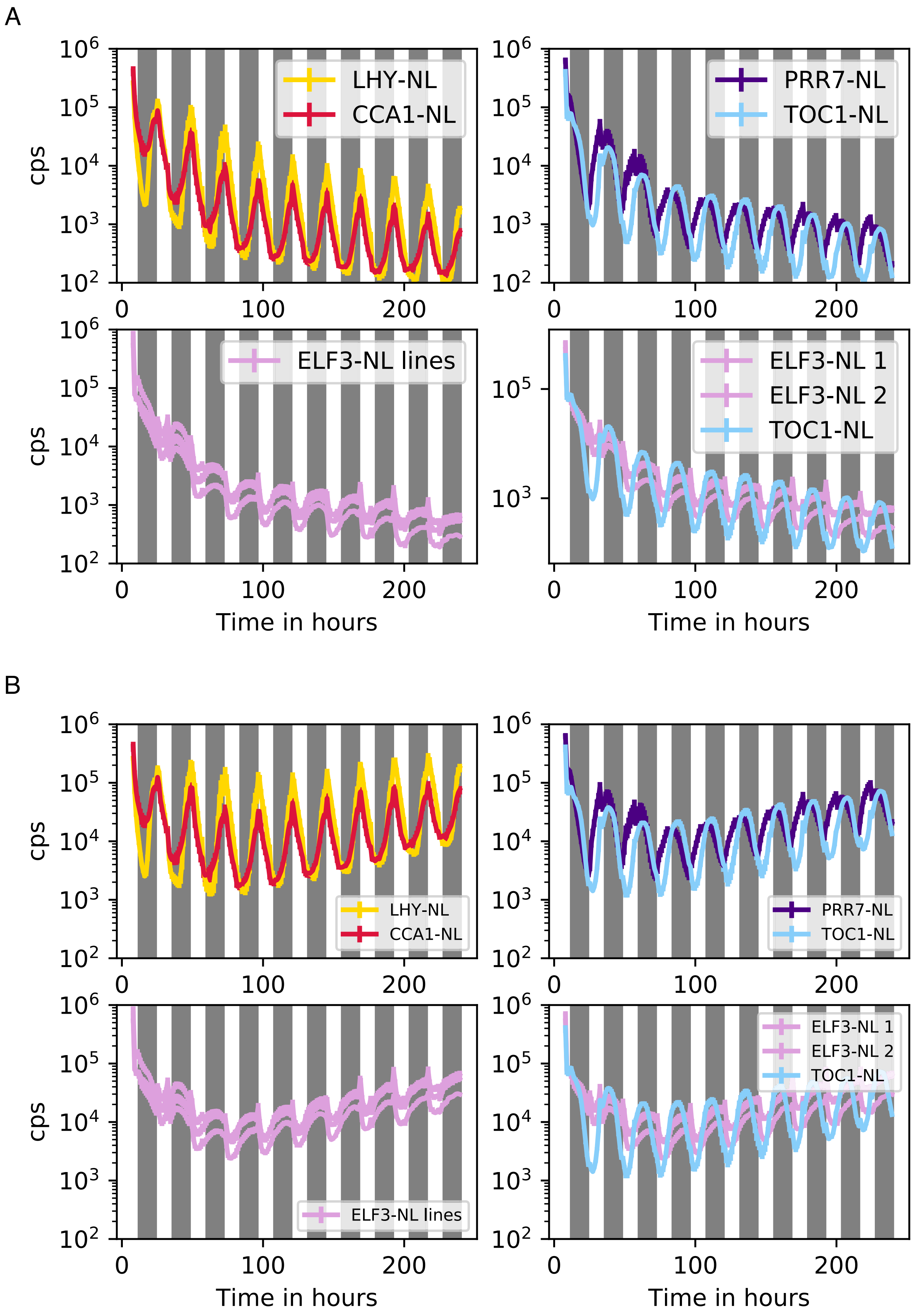
A
LHY-NL
CCA1-NL
PRR7-NL
TOC1-NL
ELF3-NL lines
ELF3-NL 1
ELF3-NL 2
TOC1-NL
cps
Time in hours
B
LHY-NL
CCA1-NL
PRR7-NL
TOC1-NL
ELF3-NL lines
ELF3-NL 1
ELF3-NL 2
TOC1-NL
cps
Time in hours

 © The Author(s)

Figure EV3. Long-term monitoring of protein fusions in vivo.

(**A**) NanoLUC activity was measured (as in Fig. 6D, E) over ten 12 L:12D cycles, in seedlings carrying the protein reporters indicated, for LHY (yellow), CCA1 (red), PRR7 (purple), TOC1 (cyan) and in three ELF3 reporter lines (pink), hourly in an automated luminometer. Each trace is from a micro-well plate seeded with 4 seeds per well and incubated under ten, 12L:12D cycles, treated with furimazine substrate and assayed for a further 10 cycles. (**B**) The falling trend due to furimazine decay was removed from the in vivo signals. A single exponential decay rate was estimated from a large data set of the CCA1 protein reporter and applied to detrend all the datasets. All the lines show acute responses to light-dark transitions.

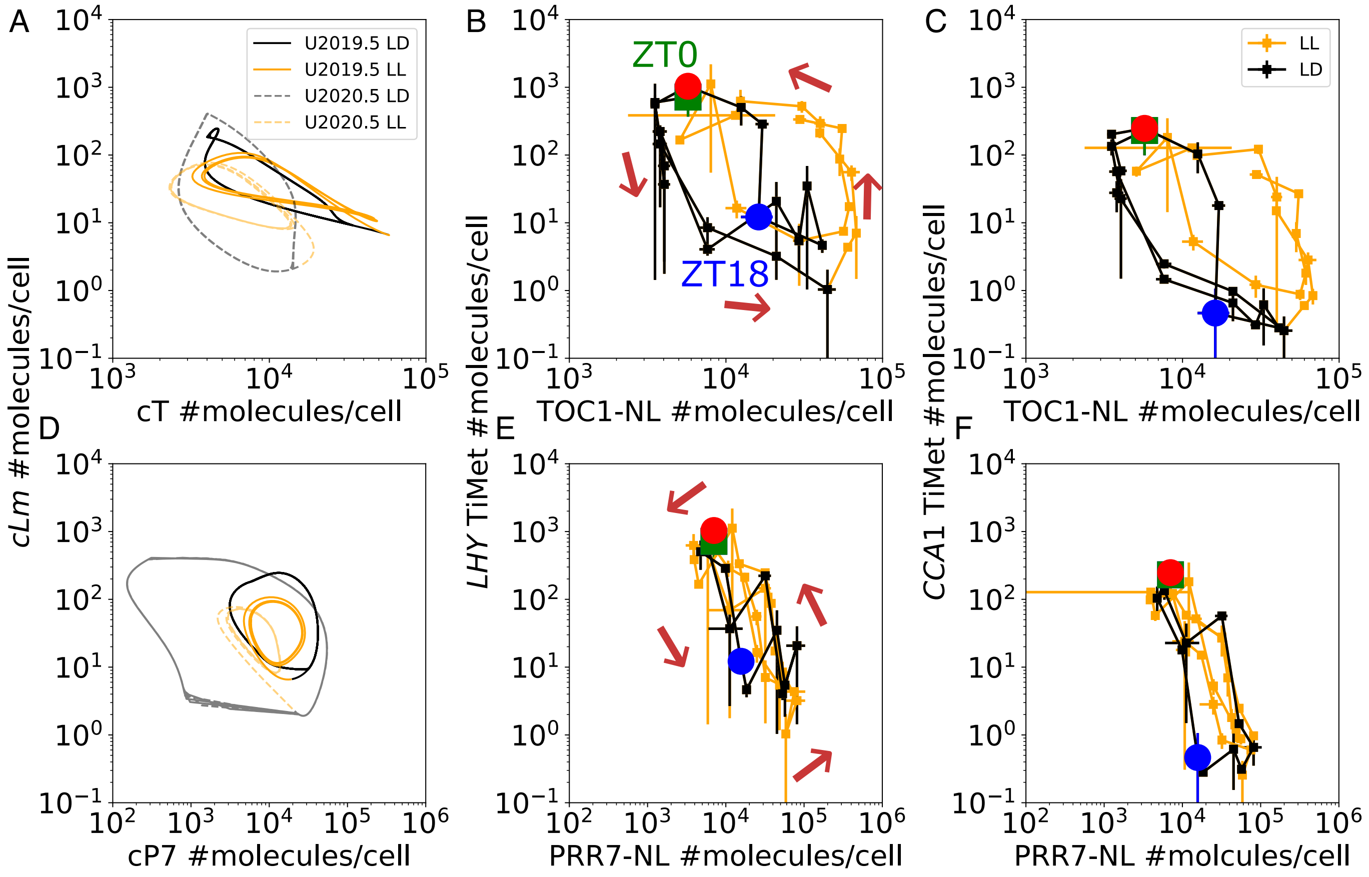

Figure EV4. Regulation of *LHY* and *CCA1* by TOC1 and PRR7.

Phase plane diagrams compare the accumulation of PRR transcriptional repressor proteins compared to their target mRNAs (as in Fig. 7). (**A**) Variables *cT* and *cLm* in models U2019.5 (dashed lines) and U2020.5 (solid lines), (**B**) TOC1 levels in extracts (as in Fig. 6) and TiMet *LHY* mRNA data, (**C**) TOC1 levels in extracts and TiMet *CCA1* mRNA data (Flis et al, 2015), under 12L:12D cycles (black lines) and constant light (yellow lines). TOC1 levels are lower when *CCA1* mRNA levels in 12L:12D than under LL (**C**), as for *LHY* mRNA levels (**B**). (**D**) Variables *cP7* and *cLm* in models U2019.5 (dashed lines) and U2020.5 (solid lines), (**E**) PRR7 levels in extracts (as in Fig. 6) and TiMet *LHY* mRNA data, (**F**) PRR7 levels in extracts and TiMet *CCA1* mRNA data, under 12L:12D cycles (black lines) and constant light (yellow lines). These variables are anti-correlated in the data (**E**, **F**) but plot a more circular cycle orbit in the model simulations (**D**). The simulated orbits also contract under LL (**D**), whereas both mRNA and protein amplitudes are maintained in the data (**E**, **F**). Markers in (**B**) show the first (green) and second (red) ZT0 (lights-on) and the intervening ZT18 (mid-night) under 12L:12D, and the direction of time (arrows). The last data point in black is ZT12 under 12L:12D. Error bars, 1 SEM.

 © The Author(s)

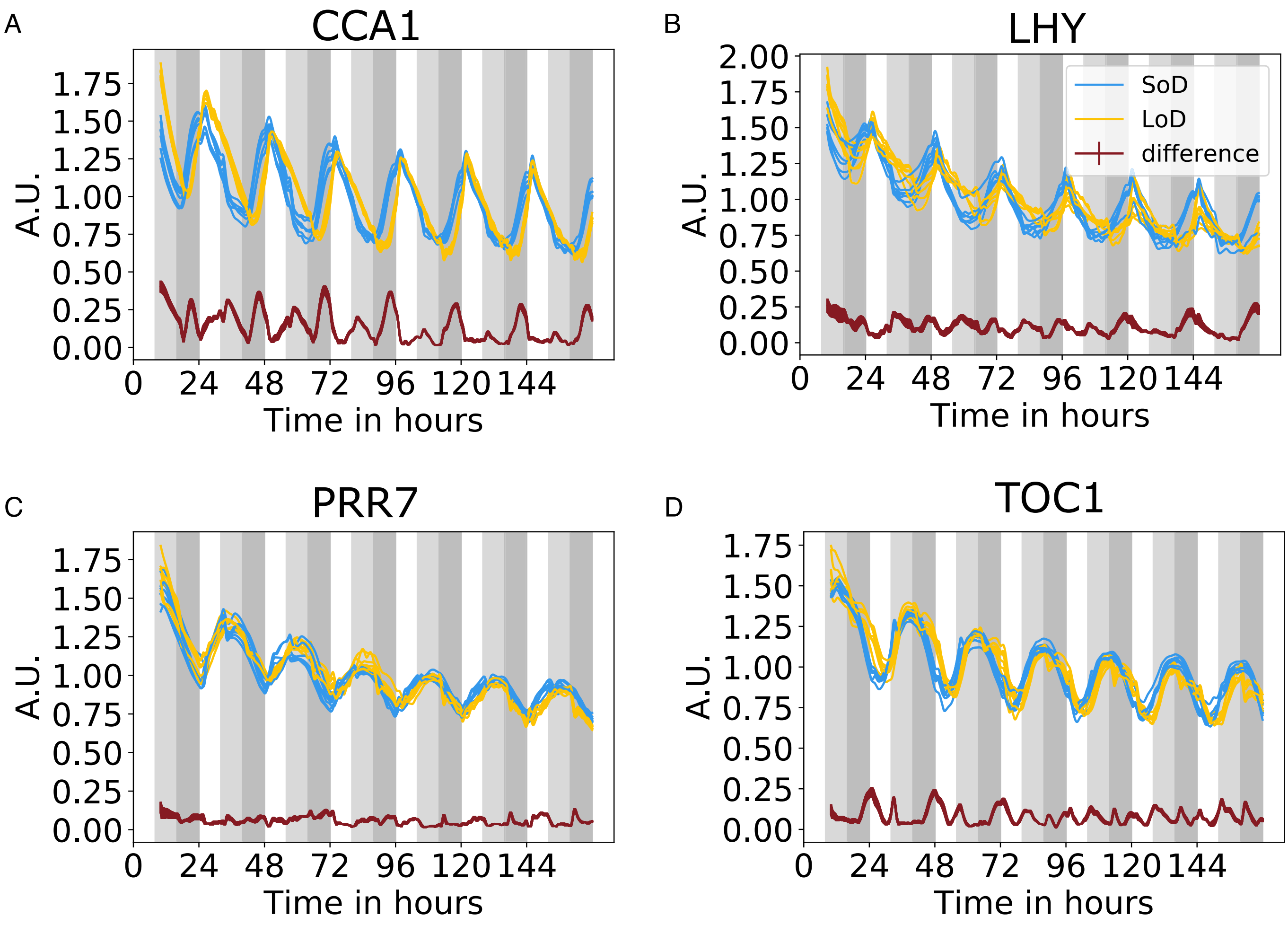

Figure EV5. Clock protein dynamics respond to photoperiod in vivo.

In vivo recording reflects expected light-responsiveness, under short (8L:16D, cyan lines; SoD) compared to long photoperiods (16L:8D, yellow lines; LoD), as in Fig. 7C. Seedlings carrying reporter protein fusions to (**A**) CCA1, (**B**) LHY, (**C**) PRR7 and (**D**) TOC1 were grown for 10 of SoD or LoD in a multi-well plate and recorded hourly for 7 days in the same conditions, using an automated luminometer. Data were log-transformed and normalised to the mean of each timeseries (giving arbitrary units, A.U.). Data points for each well were connected with a cubic spline interpolation to facilitate comparison despite slight differences in sampling times. The absolute difference between the means (red line) shows the earlier rise of expression in the night under 8L:16D. White background, light interval; light grey, dark interval in 8L:16D only; dark grey, dark in both conditions.

© The Author(s)
